# Conduct disorder and somatic health in children: a nationwide genetically sensitive study

**DOI:** 10.1186/s12888-020-03003-2

**Published:** 2020-12-17

**Authors:** Nóra Kerekes, Btissame Zouini, Emma Karlsson, Emma Cederholm, Paul Lichtenstein, Henrik Anckarsäter, Maria Råstam

**Affiliations:** 1 Department of Health Sciences, University West, Trollhättan, Sweden; 2 Department of Biology, Faculty of Sciences, Abdelmalek Essaadi University, Tetouan, Morocco; 3 Department of Surgery, Östersund Hospital, Östersund, Sweden; 4 Addiction Center, Saint Görans Hospital, Stockholm, Sweden; 5 Department of Medical Epidemiology and Biostatistics, Karolinska Institutet, Stockholm, Sweden; 6 Centre for Ethics, Law and Mental Health, Institute of Neuroscience and Physiology, University of Gothenburg, Gothenburg, Sweden; 7 Department of Clinical Sciences Lund, Child and Adolescent Psychiatry, Lund University, Lund, Sweden; 8 Gillberg Neuropsychiatry Centre, Institute of Neuroscience and Physiology, University of Gothenburg, Gothenburg, Sweden

**Keywords:** Conduct disorder, Neurodevelopmental problems, Migraine, Epilepsy, Gastrointestinal problems, Celiac disease, Lactose intolerance, Diarrhea, Constipation, Twin study

## Abstract

**Background:**

Conduct disorder (CD), a serious behavioral and emotional disorder in childhood and adolescence, characterized by disruptive behavior and breaking societal rules. Studies have explored the overlap of CD with neurodevelopmental problems (NDP). The somatic health of children with NDP has been investigated; however, the prevalence of these problems in children with CD has not been sufficiently studied. Holistic assessment of children with CD is required for establishing effective treatment strategies.

Aims: (1) Define the prevalence of selected neurological problems (migraine and epilepsy) and gastrointestinal problems (celiac disease, lactose intolerance, diarrhea, and constipation) in a population of twins aged 9 or 12; (2) Compare the prevalence of somatic problems in three subpopulations: (a) children without CD or NDP, (b) children with CD, and (c) children with both CD and NDP; (3) Select twin pairs where at least one child screened positive for CD but not NDP (proband) and map both children’s neurological and gastrointestinal problems.

**Method:**

Telephone interviews with parents of 20,302 twins in a cross-sectional, nationwide, ongoing study. According to their scores on the Autism-Tics, AD/HD, and Comorbidities inventory, screen-positive children were selected and divided into two groups: (1) children with CD Only, (2) children with CD and at least one NDP.

**Results:**

Children with CD had an increased prevalence of each neurological and gastrointestinal problem (except celiac disease), and the prevalence of somatic problems was further increased among children with comorbid CD and NDP. The presence of CD (without NDP) increased the odds of constipation for girls and the odds of epilepsy for boys. Girls with CD generally had more coexisting gastrointestinal problems than boys with CD. Female co-twins of probands with CD were strongly affected by gastrointestinal problems. Concordance analyses suggested genetic background factors in neurological and gastrointestinal problems, but no common etiology with CD could be concluded.

**Conclusion:**

Co-occurring NDP could explain most of the increased prevalence of somatic problems in CD. Our results raise a new perspective on CD in children and adolescents; their CD seems to be linked to a number of other health problems, ranging from neurodevelopmental and psychiatric disorders to somatic complaints.

## Background

Conduct disorder (CD), defined as a repetitive and persistent pattern of aggressive, defiant or antisocial behavior [1, 2], is one of the most challenging and intractable mental health problems in children and adolescents. When it persists, CD is one of the strongest risk factors for the development of antisocial personality disorder [3], criminality (especially recidivistic, violent and severe crimes) [4] and substance abuse [5]. CD is also a strong risk factor for anxiety and depression in adulthood [6, 7]. While environmental factors are known to contribute to the development of CD, the genetic components seem to be of moderate importance in boys and relatively low importance in girls [8]. CD commonly coexists with neurodevelopmental problems (NDP) such as attention deficit hyperactivity disorder (ADHD) and learning disorder (LD) [8–10]. There is some evidence of comorbidity between CD, oppositional defiant disorder (ODD) [8] and autism spectrum disorder (ASD) [11].

The co-occurrence of physical and psychiatric problems is common. In children with NDP [12, 13], ADHD [14], and ASD and/or LD [15–18] comorbidity with neurological conditions, such as epilepsy, have been described. Another neurological disorder that is often comorbid with NDP is migraine [12–14]. Previous studies have also indicated a higher frequency of gastrointestinal (GI) problems among children with NDP [13, 19, 20], including ADHD [21], ASD [22, 23] and LD [24]. The association between celiac disease and NDP in children and adolescents is inconclusive [25–27].

For children with CD, the presence of physical comorbidity patterns is less thoroughly described. Most studies did not find a significant increase in risk for aggressive behavior in children with uncomplicated epilepsy when compared with children in the general population [28], while some have indicated an increased prevalence of CD (12.5%) in children with epilepsy [29]. While migraine is common, affecting approximately 8% of children and adolescents - most commonly girls - [30], in their review of seven studies, Bruijn and colleagues [31] concluded that overall there is no evidence that migraine is associated with CD. However, Egger and colleagues [32] found that boys, but not girls, with headaches had doubled odds of having CD. This gender-specific association could not be confirmed in a clinical sample of children with pediatric migraine, but the presence of migraine increased the prevalence of disruptive behavioral problems, like ODD, generally [33]. Association between conduct and GI problems has been found in children and adolescents with ASD [34], but to our knowledge, no study on the prevalence of GI problems in children with only CD has been published.

In the present study we aimed to define the prevalence of two neurological problems, namely migraine and epilepsy, and four GI problems, namely celiac disease, lactose intolerance, constipation and diarrhea, in a nationwide general population of twins aged 9 or 12. Further, we compared the prevalence of these somatic problems in the subpopulations of children with CD with or without the coexistence of NDP. Finally, we created maps on an individual level of the somatic health of children with CD only (no NDP) and in their co-twins.

## Methods

### Screening for somatic and mental health problems

The Child and Adolescent Twin Study in Sweden (CATSS) is a longitudinal, nationwide database on twins’ somatic and mental health during childhood and adolescence. For a detailed description of CATSS, see the overview article by Anckarsäter and colleagues [35]. The substudy CATSS 9/12 was launched in 2004, collecting data on children’s mental and somatic health through parental participation in a telephone interview. The present study utilizes CATSS 9/12 data between 2004 and 2014 in a cross-sectional design. In connection with their children’s 9th birthday (or 12th birthday during only the first three years of the study), all Swedish parents of twins (identified by the Swedish Twin Registry) were asked to answer questions about their children. The response rate was 72.9%. The telephone interview typically takes about half an hour per child. Among other questions, the parents are asked if their child has ever had problems with migraine, epilepsy, celiac disease and/or lactose intolerance, prolonged periods of constipation or diarrhea. All the questions can be answered as “*yes*” (coded as 1 point), “*no*” (coded as 0 points), or “*do not know,*” or “*do not want to answer*” (coded as missing values).

In addition to direct questions about the child’s current and past somatic problems, the interview includes the Autism-Tics, AD/HD and other Comorbidities (A-TAC) inventory, a validated screening questionnaire [36–39]. The A-TAC inventory was specifically developed for the CATSS to target the main clinical diagnostic criteria of child and adolescent psychiatry through a telephone interview. The items of the A-TAC inventory can be answered “*yes*” (coded as 1 point), “*yes, to some extent*” (coded as 0.5 points) or “*no*” (coded as 0 points). The questions are worded so that the respondent parents always consider each twin separately, from a lifespan perspective.

Information from the A-TAC inventory was used to screen for children with conduct disorder (CD), attention deficit hyperactivity disorder (ADHD), autism spectrum disorder (ASD), and learning disorder (LD). The module of the A-TAC that is used for the detection of CD includes five questions, the module for ADHD includes 19 questions, the module for ASD includes 17 questions, and the module for LD three questions. The sensitivity and specificity of the A-TAC scores for predicting earlier or later clinical diagnoses were mostly good to excellent, with the following values of the area under the curve (AUC) for clinical diagnosis: 0.93 for ADHD, 0.98 for ASD, and 0.92 for LD, with small differences in terms of previous predictive analyses [39].

For the corresponding research proxy of CD, a cut-off of ≥2 was previously identified, combined with low sensitivity (0.55) and very high specificity (0.98) [8]. To be able to screen with higher sensitivity for children with CD in the present study, we used a low cut-off (score ≥ 1) on the CD module of A-TAC. This cut-off is coupled to a still high specificity (0.95) and a higher sensitivity (0.84) than the cut-off of ≥2. The sensitivity and specificity of the low cut-off (CD score ≥ 1) were determined with the use of A-TAC data from a clinical population of adolescents – previously described in Kerekes and colleagues [8 (page 4)] – in relation to the control group. The CD scale in that validation study showed an excellent overall predictive ability in receiver operating characteristics (ROC) analyses (AUC = 0.95).

For the screening for children with NDP, low cut-offs on the relevant scales were used. The ADHD scale of A-TAC contains two modules measuring concentration/attention and activity/impulsivity with nine and ten items, respectively, with a maximum of 19 points. The low cut-off score of ≥6 on the ADHD scale yields a sensitivity of 0.64 and a specificity of 0.78, as previously described by Larson and colleagues [38]. The ASD scale of the A-TAC consists of 17 items distributed between three modules covering language impairments (6 points), social interaction problems (6 points) and flexibility problems (5 points). The low cut off of ≥4.5 points for screening for children with ASD was coupled to a sensitivity of 0.70 and a specificity of 0.93 in a previous validation study [38]. Finally, the third NDP screened for in the present study was LD. The LD scale was short, consisting of three items, and its cut-off of ≥1 point was described to have a sensitivity of 0.78 and a specificity of 0.64 [38]. The psychometric properties of these NDP scales have been reported in previous studies, showing good to excellent internal and external validity [35, 38].

### Study population

With a response rate of 72.9%, the total study population included 20,302 children. Systematic analyses for the description of differences between non-responders and responders were performed in 2010 [35]. To quote that paper, “Non-responders to the CATSS 9/12 telephone interviews were more likely than responders to have: a parent treated in psychiatric settings (9.6% of the non-responders vs. 6.3% of the responders), a father convicted of a felony (11.2% vs. 7.2%), a mother convicted of a felony (1.6% vs. 0.7%), a divorced mother (16.4% vs. 12.5%), a divorced father (16.4% vs. 12.4%), or to belong to low socioeconomic strata (26.6% vs. 21.9%). Non-responders to the telephone interviews also had 2.1% ADHD as compared to 1.6% among responders, 0.95% ASD versus 0.84%, 2.0% LDs versus 0.99%. Among non-responders, 1.8% had been prescribed psychopharmacological treatment for ADHD as compared to 1.4% of the responders” [35].

**Fig. 1:**
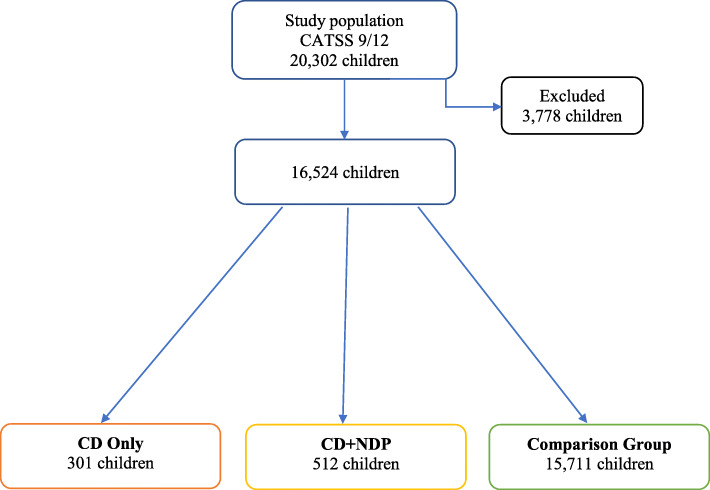
Flow chart of study groups. CATSS = Child and Adolescent Twin Study in Sweden; CD = Conduct disorder; NDP = Neurodevelopmental problems

In the study population of 20,302 children, the gender ratio was close to 1:1 with 10,344 boys (51%) and 9958 girls (49%). Of these children, 5386 (26.5%) of the twin pairs were monozygotic (MZ), 7000 (34.5%) were dizygotic same-sex (DZss), 7022 (34.6%) were dizygotic different-sex (DZds), and 894 (4.4%) had unknown zygosity. The zygosity of the twins was determined using a panel of 49 single nucleotide polymorphisms based on the children’s saliva samples; when DNA was not available, a previously validated questionnaire with 95% accuracy was used for this purpose [40]. There were 3778 children (18.6% of the study population) excluded from the analyses based on missing answers on any of the items relevant for the study (such as questions belonging to domains of ADHD, ASD, LD, CD, or missing answer on any of the somatic complaints). The most frequently missing answer (in 761 cases) was whether the child ever had epilepsy. 64.8% of the excluded children was 9-year old, and 59.9% were a boy. Keeping in mind the varying response rates: 42.6% reached the low cut-off for ADHD; 12.5% for ASD; 75.7% for LD; and none for CD. The distribution of the children included in the study is visualized on a flow chart (Fig. 1) and described below.

### Groups

A CD Only group was selected subject to the criteria of scoring ≥1 within the CD domain of A-TAC, while not reaching the cut-offs for the ADHD, ASD, or LD domains. The CD Only group consisted of 301 children; 176 (58.5%) boys and 125 (41.5%) girls; 210 (69.8%) 9-year-olds and 91 (30.2%) 12-year-olds. Of these children, 78 (25.9%) were MZ, 106 (35.2%) were DZss, 106 (35.2%) were DZds, and 11 (3.7%) had unknown zygosity.

The CD Only children were selected as probands for further descriptive analyses, specifically looking at the prevalence of somatic problems in this group compared to their co-twins.

A CD + NDP group was selected subject to the criteria of scoring at least one point in the CD domain and scoring at least on or above the cut-offs for the ADHD and/or ASD and/or LD domains. The CD + NDP group consisted of 512 children; 339 (66.2%) boys and 173 (33.8%) girls; 354 (69.1%) 9-year-olds and 158 (30.9%) 12-year-olds. Of these children 123 (24.0%) were MZ, 199 (38.9%) were DZss, 164 (32.0%) were DZds, and 26 (5.1%) had unknown zygosity.

A comparison group (CG) included all twins from the nationwide study population who did not fulfill the criteria for being included in either the CD Only group or the CD + NDP group. The CG consisted of 15,711 children; 7572 (48.2%) boys and 8139 (51.8%) girls; 10,765 (68.5%) 9-year old, 4946 (31.5%) 12-year old.

Groups are presented in Fig. 1.

### Statistical methods

The statistical analysis was conducted using SPSS version 23. Calculations were made for the prevalence of the selected somatic problems in the whole study population (CATSS) and in the groups of CD Only, CD + NDP, and CG. The odds ratios (*OR*) and confidence intervals (*CI*) for the somatic problems were calculated for children in the CD Only and CD + NDP groups. The significance was estimated by Pearson chi-square and defined at *p* < 0.05. *Cramer’s V* post-test was used to determine the strengths of association and to indicate the effect size. *Cramer’s V* value between 0 and 0.05 indicates a very small; 0.05 and 0.1 a small; 0.1 and 0.15 a medium; and greater than 0.15 indicate a strong effect size [41].

### Ethical considerations

The CATSS-9/12 study was authorized by the Ethical Review Board of Karolinska Institutet (Dnr 03–672 and 2010/507–31/1).

## Results

### Prevalence of neurological and GI problems in the general population of twins

The total population of twins (CATSS) at ages 9 or 12 consisted of 20,302 children. Out of the total population, 728 children (3.6%; 3.8% boys, 3.5% girls) were reported to have migraine and 168 children (0.9%; 1.0% boys, 0.8% girls) to have epilepsy. Both of these neurological problems had a somewhat increased prevalence in boys (*p* = 0.26 for migraine and *p* = 0.12 for epilepsy), while the prevalence of celiac disease (1.1%; 0.8% boys, 1.4% girls) was significantly higher in girls (*p* < 0.001, *Cramer’s V* = 0.03). Constipation was the most prevalent somatic problem (affecting 1531 children, 7.6%) and was more frequently reported in girls (6.3% boys, 8.9% in girls, *p* < 0.001, *Cramer’s V* = 0.05. Lactose intolerance, reported in 5.5% of the general population of twins (5.8% boys, 5.2% girls, *p* = 0.07), and prolonged periods of diarrhea, reported in 3.3% of the general population (3.9% boys, 2.7% girls, *p* < 0.001, *Cramer’s V* = 0.03) were more frequent problems in boys.

### Prevalence of somatic problems in the defined subpopulations

#### CD only group

**Fig. 2:**
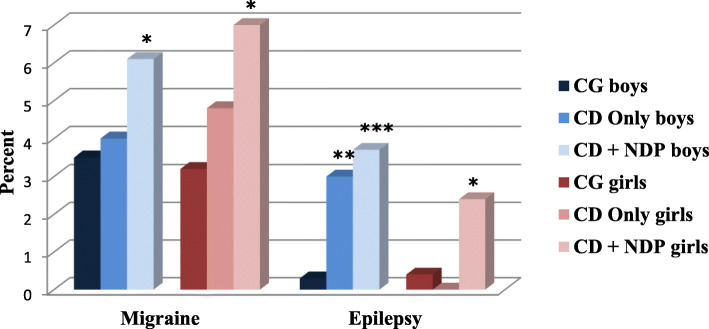
Prevalence of children with reported neurological problems (migraine and epilepsy). CG = Comparison group excluded those with CD Only and CD + NDP. CD = Conduct disorder; NDP = Neurodevelopmental problems

**Fig. 3:**
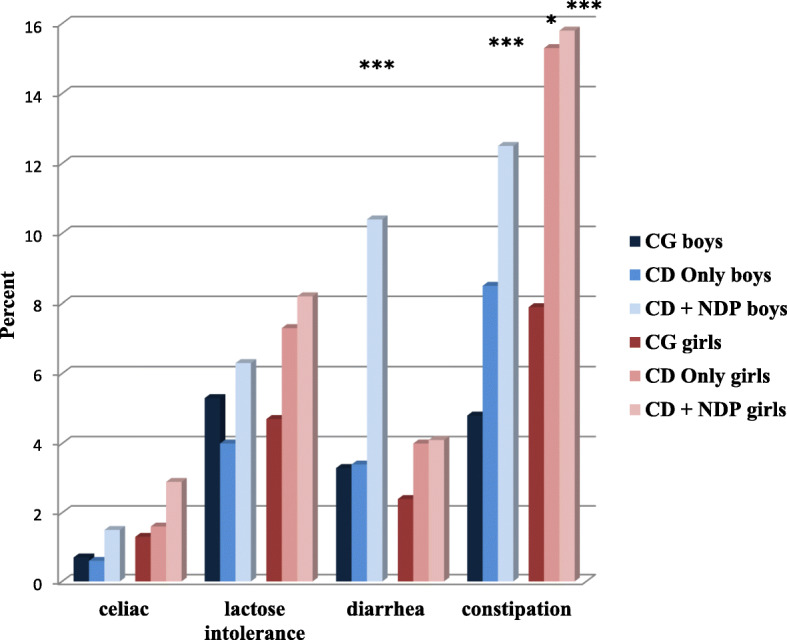
Prevalence of children with reported GI problems (celiac disease, lactose intolerance, diarrhea, and constipation). CG = Comparison group excluded those with CD Only and CD + NDP. CD = Conduct disorder. NDP = Neurodevelopmental problems

In the subpopulation of children belonging to the CD Only group (*n* = 301), the prevalences of both neurological problems (migraine and epilepsy) (Fig. 2) and two of the GI problems (diarrhea and constipation) were increased (Fig. 3), but most of these differences did not reach statistical significance, except the prevalence of epilepsy and constipation which were increased significantly (*p* < 0.001) with a small effect size (Table 1). The analysis of differences between boys and girls gave more distinct patterns. The odds for a CD Only girl to also have migraine, lactose intolerance, or diarrhea were increased, but not significantly (with 42, 45, and 51%, respectively) (Table 1). The odds that a CD Only girl had constipation was significantly increased (*p* < 0.001), with 88%, compared to a CG girl (Table 1). In CD Only boys, the odds of having epilepsy increased almost three and a half times, highly significantly (*p* < 0.001) with 246%, while the odds of having constipation was increased (41%) but not significantly (Table 1).

**Table 1:** Prevalence and odds ratio of somatic problems in the nationwide population of 9- or 12-year-old twins

	CG % (Boys/ girls) *N* = 15,711	CD Only % (Boys/ girls) *n* = 301	*P-value* *(Cramer’s V)*	CD + NDP % (Boys/ girls) *n* = 512	*P-value* *(Cramer’s V)*	CD only *OR* (Boys/girls) [CI]	CD + NDP *OR* (Boys/girls) [CI]
Migraine	3.4 (3.5/3.2)	4.3 (4.0/4.8)	0.3	6.4 (6.1/7.0)	< 0.001 (0.03)	1.21 (1.06/1.42) [0.69–2.12]	1.85*** (1.68*/2.13*) [1.28–2.67]
Epilepsy	0.4 (0.3/0.4)	1.7 (3.0/0.0)	< 0.001 (0.03)	3.2 (3.7/2.4)	< 0.001 (0.07)	2.16 (3.46**/0.99) [0.88–5.30]	4.45*** (4.75***/3.8*) [2.63–7.52]
Celiac disease	1.0 (0.7/1.3)	1.0 (0.6/1.6)	0.94	2.0 (1.5/2.9)	0.05	0.90 (0.68/1.14) [0.29–2.81]	1.82 (1.86/2.13) [0.96–3.45]
Lactose intolerance	5.0 (5.3/4.7)	5.4 (4.0/7.3)	0.78	6.9 (6.3/8.2)	0.05	0.98 (0.68/1.45) [0.59–1.62]	1.30 (1.10/1.67) [0.92–1.84]
Diarrhoea	2.8 (3.3/2.4)	3.7 (3.4/4.0)	0.39	8.3 (10.4/4.1)	< 0.001 (0.06)	1.11 (0.86/1.51) [0.60–2.03]	2.72*** (3.01***/1.54) [1.97–3.77]
Constipation	6.4 (4.8/7.9)	11.3 (8.5/15.3)	< 0.001 (0.05)	13.6 (12.5/15.8)	0.001 (0.03)	1.58* (1.41/1.88*) [1.10–2.27]	1.97*** (2.22***/1.95***) [1.52–2.56]

* *p* < 0.05, ** *p* < 0.01, *** *p* < 0.001

CG = Comparison group, excluded those with “CD Only” and “CD + NDP”

CD = Conduct disorder; NDP = Neurodevelopmental problems; *OR* = Odds ratio

#### CD + NDP group

Figures 2 and 3 and Table 1 summarize information about the prevalence and *OR* of the defined somatic problems in the subpopulation of children with CD and NDP (512 children). The prevalence of migraine in these children was 6.4% (*n* = 32), significantly higher (*p* < 0.001), with a small effect size, than in CG. There was a significant increase (*p* < 0.05) of the odds of migraine in both genders.

The prevalence of epilepsy (16 children; 3.2%) in the CD + NDP group was also significantly higher (*p* < 0.001) with a small effect size, than in the CG. The gender distribution was uneven: the odds for a CD + NDP boy to also have epilepsy increased almost five times (*p* < 0.001), while for a girl with the same conditions, the odds increased almost four times (*p* < 0.05).

Generally, the coexistence of CD and NDP in a child further increased the odds of co-occurring GI problems in both genders. The prevalence of celiac disease and its odds almost doubled, but without reaching statistical significance, whilst mainly affecting girls. The odds of lactose intolerance increased (but not significantly) more for CD + NDP group girls than for boys in the same group. The prevalence of diarrhea (*p* < 0.001) and constipation (*p* = 0.001) was significantly increased with a small effect size in CD + NDP children (8.3% for diarrhea; 13.6% for constipation). The odds of problems with prolonged periods of constipation for these children were doubled (*p* < 0.001) for both genders, and the odds for problems with prolonged periods of diarrhea tripled (*p* < 0.001) for CD + NDP boys.

### Maps of somatic problems in CD only children and their co-twins

In the concordance analysis of the 301 children belonging to the CD Only group, one child (DZss) was excluded due to data missing about his co-twin. In 25 cases, both twins belonged to the CD Only group, in which case the twin with the highest score in the CD domain was chosen as proband. The CD Only group included a total of 275 probands. A total of 61 children (35 boys and 26 girls) were MZ, 99 (63 boys and 36 girls) were DZss, and 104 (59 boys and 45 girls) were DZds, while 11 probands with unknown zygosity were excluded at this step of the study.

For the prevalence of the different somatic problems in CD Only children, please see Table 1. When investigating the concordance rate for the somatic problems in the general population, we found that MZ twin pairs were at least twice as often concordant for the specific problem than DZ pairs in each problem area (Table 2). In the CD Only group, no MZ twin pair was concordant for any of the investigated somatic problems, except for constipation, where the concordance rate was more than doubled in these MZ twin pairs compared to DZ twin pairs (Table 2).

**Table 2:** The concordance rate of defined somatic problems in MZ and DZ twin pairs

	CATSS general population			CD Only		
	MZ (*nr* Cc:Dc)	DZss (*nr* Cc:Dc)	DZds (*nr* Cc:Dc)	MZ (*nr* Cc:Dc)	DZss (*nr* Cc:Dc)	DZds (*nr* Cc:Dc)
Migraine	21.88% (35:125)	7.35% (10:126)	3.31% (8:234)	-% 0:2	12.5% 1:7	-% 0:7
Epilepsy	13.89% (5:31)	6.56% (4:57)	6.25% (3:45)	-% -:-	-% 0:6	-% -:-
Celiac disease	66.67% (24:12)	19.12% (13:55)	10.94% (7:57)	-% -:1	-% -:1	-% -:-
Lactose intolerance	50.27% (94:93)	19.09% (59:250)	18.29% (60:268)	-% 0:2	16.67% 1:5	18.75% 3:13
Diarrhea	35.77% (44:79)	8.29% (17:188)	7.59% (17:207)	-% -:2	50% 1:1	8.33% 1:11
Constipation	23.94% (62:197)	4.66% (25:512)	6.11% (33:507)	57.14% 4:3	-% 0:24	4.76% 1:20

*MZ* monzygote, *DZss* dizygote same-sex, *DZds* dizygote different-sex, *nr Cc:Dc* number of concordant twin pairs and nr of discordant twin pairs

The mapping of each CD Only twin pair’s somatic problems and indicating the proband severity of CD showed no association between the number of CD points (severity) and the number of somatic complaints (*Spearman r* = 0.027; *p* = 0.67).

**Fig. 4:**
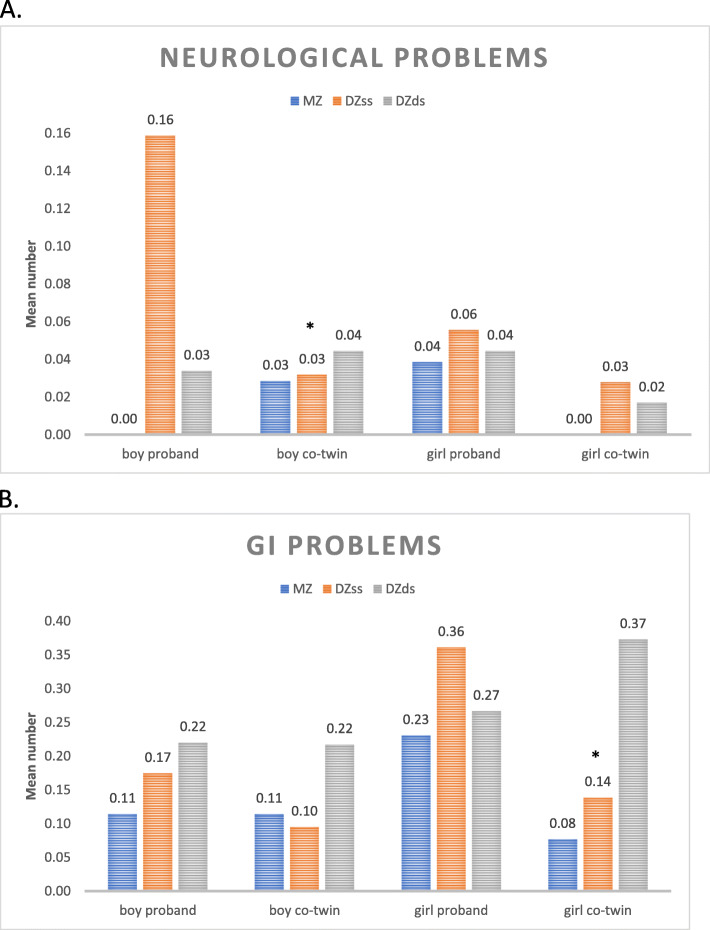
**a-b**: Mean number of somatic problems in CD Only children (probands) and in their co-twins. (**a**) Neurological problems (**b**) Gastrointestinal problems. MZ = monzygote, DZss = dizygote same-sex, DZds = dizygot different-sex. * *p* < 0.05

The calculation of the mean number of somatic problems per proband and co-twin separately for boys and girls showed, as previously indicated, that proband boys had more neurological problems than proband girls (Fig. 4a), and that proband girls had more GI problems than proband boys (Fig. 4b). The mean numbers of somatic problems were lower in both boy and girl co-twins than in their probands (except neurological problems in MZ boys, which was not reported in probands) (Fig. 4a). This difference reached a significance level for neurological problems in DZss boys (*p* = 0.027) and for GI problems in DZss girls (*p* = 0.03) (Fig. 4a and b). It is noteworthy that DZss proband girls and DZds co-twin girls were the most strongly affected by GI problems (Fig. 4b). These girls had a higher frequency of somatic problems regardless of whether or not they had been screened positive for CD.

## Discussion

### Main findings

The prevalence of somatic problems was increased in children with CD Only. In children with both CD and NDP, the odds of neurological problems (migraine and epilepsy) and/or GI problems (celiac disease, lactose intolerance, diarrhea, and constipation) were even more increased.

Gender-specific patterns of coexisting somatic problems were found. In both children with CD Only and children with both CD and NDP, the odds of migraine was increased more in girls, and the odds of epilepsy was increased more in boys. Generally, girls having screened positive for CD with or without NDP had more coexisting GI problems than boys in the same groups.

The concordance rates suggest genetic background factors behind each investigated neurological and GI problem, though we found no evidence linked to CD as such.

Co-twins of probands belonging to the group of children with CD Only did not have an increased odds of neurological problems. However, female twins were strongly affected by GI problems.

### Neurological and GI problems in the general population of twins

The relationship between neurological and GI problems is well known, as several studies have shown that GI problems coexist with neurological diseases, and neurological complications may become evident with GI diseases [42, 43]. For example, GI disorders, e.g., vomiting or chronic diarrhea, may cause electrolyte imbalances [44], or they may, e.g., inflammatory bowel disease, be coupled to thromboembolic events or vitamin B12 and thiamine deficiency, and be accompanied with epilepsy [45]. Doulberis and colleagues [46] recently published a comprehensive review of the relevant studies linking migraine and GI-related disorders. The investigators found a clear association between migraine and various GI diseases, including irritable bowel syndrome, inflammatory bowel disease, celiac disease, Helicobacter pylori infection, and cyclic vomiting syndrome, as well as food allergy and infantile colic [46].

In the following, we discuss the results according to the aim of the study, such as (1) define the prevalence of neurological problems and GI problems in a nationwide general population of twins aged 9 or 12; (2) compare the prevalence of these somatic problems in the subpopulations of children with CD with or without the coexistence of NDP; (3) map on an individual level the somatic health of children with CD Only and in their co-twins.

#### Migraine

A systematic review of population-based studies estimated the prevalence of migraine in children and adolescents (under 20 years) to 7.7% (ranging between 0.5 and 21.7%), with a higher prevalence in girls compared to boys [30]. The prevalence of migraine in our study was 3.6% in the population of 9- or 12-year-old Swedish children, and we could not confirm a higher prevalence of migraine in girls than in boys. The reasons for these discrepancies could be that our study population’s age was in the lower range compared to those reviewed previously [30] and that many of the reviewed studies collected data from children and adolescents who were actively seeking help for headaches. Our study design made it possible to investigate the concordance rates of migraine in siblings. We found a more than five times higher concordance in MZ twin pairs than in DZss twin pairs. This observation suggests a substantial genetic influence behind the development of migraine, which is in line with some earlier studies that have shown genetic components as risk factors for developing migraine [47, 48].

#### Epilepsy

The prevalence of epilepsy in the nationwide population of 9- or 12-year-old twins was 0.9%, with a slightly higher prevalence in boys than girls. This is a comparable result with that published in the latest review on the prevalence of epilepsy, where the average prevalence of epilepsy in children aged 10 to 19, based on 12 studies, was 0.89% [49]. Using the genetically sensitive study population, we could calculate concordance rates for epilepsy that showed a more than twice increased concordance rate in MZ twin pairs than DZss twin pairs, thus confirming earlier findings that epilepsy has a strong genetic component [50].

#### GI problems

Some degree of GI problems is relatively common in children in the general population. In our study, reports of celiac disease were found for 1.1% of the children, which corresponds well with previous studies on the seroprevalence of celiac disease worldwide [51]. A closer comparison to previous Scandinavian studies shows similarities [52] and differences [53]. Lactose intolerance was found in 5.5% of the children in the present study. This prevalence is lower than that found in a previous Swedish study, where parents reported a lactose intolerance in 9% in 8-year-olds, and 13% in 12-year-olds children [54]. The prevalences of diarrhea (3.3%) and constipation (7.6%) in our study are slightly lower than in the review of Kokkonen and colleagues [55] in Finland, which found prevalence figures of 6 and 9%, respectively.

It is important to note that diarrhea and constipation are often symptoms of other specific diagnoses, whereas celiac disease and lactose intolerance are diagnoses in themselves. For instance, diarrhea and constipation are common in celiac disease [56], and diarrhea is commonly present in children with lactose intolerance [57].

We detected gender-specific patterns of GI problems in the general sample. Celiac disease was more prevalent in girls than in boys, which confirmed earlier reports of this difference in prevalence [58]. Our finding of a significantly higher incidence of constipation in girls than in boys confirmed the reports in two of seven studies in a systematic review, whereas the other five studies did not show a gender difference [59]. In contrast, the findings in our study that boys are possibly more prone to have lactose intolerance (no significant difference between genders) and significantly more often have complaints of diarrhea, compared to girls, are not generally supported in the literature. For example, Winberg and colleagues [54] reported that lactose intolerance is more common in Swedish girls than in boys (*OR* 1.7). For chronic diarrhea, it is difficult to compare prevalence figures, given that it is a symptom with several potential causes, including infections and antibiotics treatment, irritable bowel syndrome, lactose intolerance, and celiac disease for older children [60]. The most commonly described functional cause of chronic diarrhea in children is irritable bowel syndrome [60], and a female predominance has been described [61].

### Prevalence of somatic problems in children with CD only or with both CD and NDP

Because there is a significant overlap of CD with ADHD, ASD, and LD [9, 10, 62], we created a group of children with ‘purely’ aggressive behavioral problems, the CD Only group, by excluding all children with coexisting NDP (ADHD, ASD, and LD). Moreover, we created a CD + NDP group, including children with both CD and NDP.

#### Neurological problems

When focusing on neurological problems in these two groups, we found a slightly higher prevalence of migraine in children belonging to the CD Only group, which showed no significant increase in odds compared to the comparison group. In the children who had NDP comorbid with CD, the prevalence of migraine was significantly increased, and the odds doubled in both genders, with a greater effect on girls. This result confirmed previous research findings showing an increased prevalence of migraine in children with NDP [13, 33]. The results indicate that the behavioral problem (CD) itself does not increase the odds of having migraine; the odds increases only when CD is combined with NDP.

Epilepsy has been described to be more prevalent in children with NDP, especially in those with LD; approximately one in four children with moderate to severe LD has epilepsy [15]. Epilepsy is also more common in children with ADHD and/or ASD than in the general population [13, 18, 63]. It has been suggested that the reasons for this could be structural and/or functional differences in certain areas of the brain, as certain types of epilepsy are more common (frontal lobe epilepsy, childhood absence epilepsy, and Rolandic epilepsy) in children with NDP [63]. The prevalence of epilepsy in the CD Only group was twice that of the prevalence found in the comparison group, with the increased OR reaching significance in boys. When including NDP in the psychopathology, the prevalence of epilepsy further increased by almost five times in boys and 3.5 times in girls. The increased odds of epilepsy seem to be gender-specific in our sample. Boys with CD Only or with both CD and NDP had much higher odds of epilepsy than girls. A possible and partial explanation for this could be the more frequent occurrence of both CD and NDP in boys in general [8, 62]. Even though we had an unusually large study population, the number of girls with CD and NDP was still low. This low prevalence makes it more challenging to find comorbidity with other low-prevalence diagnoses. Gender-specific aspects of the prevalence of epilepsy have previously been described [64].

#### GI problems

Previous studies have shown remarkable differences in the prevalence of GI problems among children with NDP, with frequencies ranging from under 5% to around 40% [21, 65]. Children with ADHD and/or ASD are often reported to have a higher frequency of GI problems than children with typical development [13, 21–23, 66]. Two of the most common GI symptoms coexisting with these NDPs are chronic diarrhea and chronic constipation [13, 21–23, 66]. A few previous studies have explored the association between NDPs and celiac disease and lactose intolerance among children [13, 27]. However, the present study is the first to focus on children with CD only and their coexisting GI problems.

When analyzing the frequencies of GI problems in the children belonging to the CD Only group, these frequencies were found to not differ from those found in children belonging to the comparison group. However, girls with CD Only had a 50% increase in lactose intolerance and/or diarrhea, and a significantly increased, almost doubled, odds of problems with prolonged periods of constipation compared to girls from the comparison group. In boys with CD Only no increased prevalence of any GI problem was detected, with the exception of a 1.4 times increase in reports of problems with constipation. These results suggest that girls with CD have more psychosomatic problems, which has been confirmed in earlier studies, such as one on functional abdominal pain [20]. It is known that girls’ disruptive behavior is usually expressed differently to that of boys. CD in girls includes more indirect and/or self-directed aggressive acts, whereas boys usually engage in direct and proactive aggression [67]. Based on our findings, it appears that girls’ indirect and introverted aggression is associated with more somatic symptoms and, thus, an increased prevalence of GI problems.

Generally, the link between CD and GI problems could be the altered serotonin levels. A decreased level of serotonin is coupled with aggression [68, 69]. Interestingly, De Theije and colleagues [69] have a theory that during an inflammatory process in the gut, serotonin is produced at higher quantities, which results in a faster bowel movement, increased secretion, and vascular permeability. This leads to a different stool pattern, with diarrhea or constipation. They also suggest that there will be less tryptophan left as a substrate for brain serotonin because of the increased use of dietary tryptophan in the gut. Therefore, they argue that the brain’s serotonin level will decrease, which may impact the person’s mood and entail cognitive dysfunctions [70].

A possible conclusion of our result and the theory mentioned above could be that if ongoing inflammatory conditions of the GI tract can be found and treated in children, then the increased metabolism of dietary tryptophan could be stopped and, consequently, the production of the brain serotonin would increase. Physiologically increased serotonin levels may result in attenuated aggression.

In the CD + NDP group, there was a further increase in the odds of each of the four GI problems investigated; however, this increase was only statistically significant for diarrhea and constipation. It has been suggested that the overlap between NDP and behavior problems is strongly associated with the severity of the problems [71]. If this is valid for our population, then we can assume that the children within this comorbid group will present more severe symptoms of both CD and NDP. This explanation accords with previous studies suggesting that children with more severe ASD have more severe GI symptoms [72]. A description of the relationship between somatic complaints and NDP in our population has been previously published [13].

### Somatic map of children with purely behavioral problems

For each investigated somatic problem, the concordance rate was at least twice as high in MZ twin pairs as in DZ twin pairs, suggesting a strong genetic effect in the development of these problems. This pattern could not be confirmed in the small group of children with CD Only. This might be explained by the low prevalence of both CD and each of the somatic problems. However, the concordance rate for constipation in the MZ twin pairs was 80%, whereas only 6% of the DZ twin pairs were concordant. Since constipation is a common symptom in children with celiac disease, this information could suggest that the affected twins have an underlying, yet undiscovered, celiac disease. The high concordance rate is very similar to that found in MZ twins for celiac disease (70%) in a previous study [56].

No correlations between the severity of CD and the number of somatic problems were found. Interestingly, girl probands, but even co-twin girls who had a CD Only brother, had the highest frequency of somatic problems, especially constipation and diarrhea. Girls with externalizing problem behaviors have low self-esteem and report psychosomatic problems more often than boys [73]. It is also possible that co-twin girls with a CD Only brother are more affected by the family situation with their brother than are co-twin boys with CD Only sisters, or that GI problems in co-twin girls are signs of not yet developed or diagnosed somatic or psychiatric disorders.

### Clinical and scientific importance

Gillberg [71] discusses the need for a multidisciplinary approach to neurodevelopmental disorders in order to be able to consider all problem areas for a patient and not just the remote diagnosis. In a previous paper, we showed that children with neurodevelopmental problems, corresponding to one or several diagnoses, face substantially increased odds also of somatic disorders [13]. Based on those data, we stressed the need for heightened awareness of possible coexisting somatic disorders in neurodevelopmental child psychiatry. The present study shows that this is also true for assessments of children with a purely behavioral disorder, such as conduct disorder. These children are often assessed in a purely social context by social services personnel, who often investigate the child’s situation with limited psychological and psychiatric resources; a comprehensive pediatric somatic evaluation is rarely part of the assessment of the child. As a minimum, it is important that trained nurses, aware of the increased risk of comorbidities, be involved in such assessments.

Our results show a considerable overlap of somatic problems in children with CD and NDP, suggesting the importance of a holistic view in pediatric diagnosis and treatment strategies. Furthermore, our results raise a new perspective on CD in children and adolescents; their conduct disorder seems to be linked to several other health problems, ranging from neurodevelopmental and psychiatric disorders to somatic complaints and pain.

### Limitations

This study has several significant limitations. The diagnoses of migraine, epilepsy, and gastrointestinal problems in CATSS 9/12 were based on parental reports and not clinical diagnoses. Parents generally have fairly good knowledge about the existence of their children’s gastrointestinal symptoms but not necessarily about the exact nature of the problem [74, 75]. It is likely that parents may report lower rates of somatic problems when their child also suffers from other more dominant problem areas, for instance, psychiatric issues.

The fact that all interviews were done by telephone is a limitation, too. However, the telephone-interview used has been validated in numerous studies by the authors and other research groups and has been found to have good psychometric properties. This is also in line with previous studies showing small differences in the assessment of psychiatric disorders made by telephone or face-to-face interviews [76–78].

While the study analyses data from a substantially large and nationwide study population, it should be considered as a limitation that among those not participating in the study (27% from the nationwide population), there was a higher prevalence of ADHD, ASD, and LD, more of them belonged to lower socioeconomic strata, and a larger proportion of them lived in families with registered criminal behavior. Some data were excluded from the analyses because of missing information from the parents on crucial variables for the present study. In the excluded subgroup of children (18.6%), there was an about four times increased prevalence of ADHD and ASD compared to the total population. The majority of the excluded children (75.5%) had LD, while none of them had CD. The prevalence of somatic complaints in the excluded subgroup was comparable with those in the CD + NDP group. Based on the high response rate (over 70%) in CATSS 9/12, and the fact that no child with CD diagnosis was excluded, we could assume that those children whose data were analyzed in the present study are generalizable considering the prevalence of somatic complaints in children with CD.

Our study’s obvious limitation is that the data describe twins, which entails that the results should be generalized with caution. However, twins have been proved to be similar to singletons when evaluating the prevalence of psychiatric and somatic problems [79–81].

Another limitation is that the investigated types of neurological and GI problems were limited. We did not consider some other neurological problems such as myasthenia gravis, encephalitis, stroke, etc.; neither considered other GI problems such as nausea, vomiting, abdominal pain, reflux, bloating, inflammatory bowel disease, etc.

Given that this is a cross-sectional study, this research establishes an association between CD and physiological symptoms but not its causality. Further research is warranted to examine symptom development and the pathological processes at play.

## Conclusion

The present, nationwide, genetically sensitive study results are significant and novel, proving the importance of the implementation of holistic thinking within psychiatry care. We found that the presence of CD, with or without NDP, increases the odds of migraine, especially in girls, and the odds of epilepsy, especially in boys. Generally, girls with behavioral problems more frequently suffer from GI problems than boys, and twin sisters of boys with disruptive behavior problems manifest more somatic complaints than twin brothers of girls with CD. Thus, in children with behavioral problems, somatic complications should be considered.

Future research should concentrate on the challenges and meaning of a holistic perspective from diagnosis to treatment. We all should see the whole person when meeting patients, not blinding our eyes from somatic complaints in psychiatry or mental health problems in medicine. Only when we will see each person as a whole can we offer individualized, equal, and effective care.

## Data Availability

The dataset supporting this article’s conclusions is available with a request to the first and corresponding author (Nóra Kerekes).

## Abbreviations

ADHD: Attention deficit hyperactivity disorder
ASD: Autism spectrum disorder
A-TAC: Autism-Tics, AD/HD and other Comorbidities
AUC: Area under the curve
CATSS: Child and Adolescent Twin Study in Sweden
CD: Conduct disorder
DZds: Dizygotic different-sex
DZss: Dizygotic same-sex
GI: Gastrointestinal problems
LD: Learning disorder
MZ: Monozygotic
NDP: Neurodevelopmental problems
ODD: Oppositional defiant disorder
